# Genomic Sequence of the New Attenuated Vaccine Strain Neethling-RIBSP of the Lumpy Skin Disease Virus

**DOI:** 10.1128/MRA.00318-20

**Published:** 2020-06-25

**Authors:** M. B. Orynbayev, R. K. Nissanova, T. U. Argimbayeva, K. D. Zakarya, B. S. Myrzakhmetova, A. M. Melisbek, S. M. Barmak, A. U. Issabek, A. K. Nakhanov, A. Shevtsov, N. S. Kozhabergenov, K. T. Sultankulova

**Affiliations:** a RGE Research Institute for Biological Safety Problems, SC/MES RK, Gvardeyskiy, Republic of Kazakhstan; b K. I. Skryabin Kyrgyz Agrarian University, Bishkek, Kyrgyz Republic; c Al-Farabi Kazakh National University, Almaty, Republic of Kazakhstan; d RSE National Center for Biotechnology, SC/MES RK, Nur-Sultan, Republic of Kazakhstan; KU Leuven

## Abstract

We report here the draft genome sequence of the new attenuated strain Neethling-RIBSP of the lumpy skin disease virus, obtained by sequential and alternating passages in cell culture and developing chicken embryos. Genome sequencing allowed the identification of differentiation markers of the new strain.

## ANNOUNCEMENT

Lumpy skin disease is an infectious disease of viral etiology caused by a DNA-containing virus of the *Capripoxvirus* genus within the *Poxviridae* family (1). The only way to successfully control disease is quick and efficient diagnosis followed by vaccination. In Kazakhstan, the disease was first registered in 2016 in the Atyrau region (2). Sequential and alternating passages in various cell cultures and developing chick embryos of the virus isolated during the above-mentioned outbreak resulted in the attenuated Neethling-RIBSP strain. Initially, 49 passages were performed in lamb testicle cell culture, followed by 5 passages in chorioallantoic membranes of embryonated chicken eggs and 33 passages in the MDBK cell line. Studies have shown that the new strain has lost its virulence, is harmless to cattle, and has pronounced immunogenic properties (3).

The current genome characterization will allow us to better understand the attenuation mechanism for obtaining vaccine strains and reveal mutations for their differentiation from the other strains. Here, we report a near-complete genome sequence of the Neethling-RIBSP strain.

DNA was extracted from a virus purified on a stepwise sucrose density gradient of 45 to 48 to 60% (4) using the DNeasy blood and tissue kit 250) (Qiagen) following the manufacturer’s protocol. The library was prepared by using the Nextera XT DNA library preparation kit. Sequencing was performed using the MiSeq reagent kit v3 (600 cycles) on the Illumina MiSeq platform. The quality of sequences was assessed using FastQC v0.11.15 (5). Genome assembly was performed using SPAdes v3.13 (6). Default parameters were used for all software unless otherwise specified. The BLAST program was used for comparative identification of the obtained contigs. A total of 574,330 paired-end reads (2 × 300 bp) were obtained, during the assembly of which 398 contigs were obtained that ranged in size from 1,000 to 146,159 bp (coverage, 2 to 517×). The longest contig, with a coverage of 59.5×, was identified as lumpy skin disease virus, and the remaining contigs were assigned to Bos taurus. A pairwise comparison analysis showed that the new vaccine strain is 99.96% identical to the Kubash/KAZ/16 strain. The missing parts are 2,143 bp long at both the 5′ and 3′ ends of the genome compared to the reference genome. Comparative analysis of the sequence of the Neethling-RIBSP vaccine strain and the Kubash/KAZ/16 strain (2019; GenBank accession number MN642592) detected 5 point substitutions and 4 insertions in the genome, as well as 3 large deletions at the gene loci (in LD007, LD016, and the noncoding region) (Table 1). The deletion in gene LD007, encoding an ankyrin repeat protein, represented the loss of a fragment of 16 nucleotides (nt), resulting in a premature stop codon and, hence, a truncated protein. An 18-nt deletion in gene LD016, encoding an Ig domain OX-2-like protein, resulted in a 6-amino-acid (aa) deletion in comparison with the Kubash/KAZ/16 strain. The third deletion showed the loss of 11 nucleotides located in a noncoding sequence enriched with thymine and adenine. The identified deletions found only in the Neethling-RIBSP strain can be used to differentiate it from other vaccines (GenBank accession numbers KX764645.1, KX764643.1, and KX764644.1) and virulent strains by sequencing and real-time PCR.

**TABLE 1:** Nucleotide modifications and their effects on the encoding sequence in Neethling-RIBSP versus the field strain Kubash/KAZ/16 (GenBank accession number MN642592)

Gene or IR^*a*^	Nucleotide modification	Change in coding sequence
LD 007	Deletion (loss of 16 nt)	Premature stop codon at position 386
IR LD 007 to LD 008	Insertion (2 nt, TT)	
LD 009	Mutation (substitution C to A)	Glycine→cysteine at position 439
LD 016	Deletion (loss of 18 nt)	6-aa deletion without amino acid changes at the breakpoint at position 141
IR LD 019 to LD 020	Insertion (1 nt, T)	
IR LD 019 to LD 020	Deletion (loss of 11 nt)	
LD 052	Mutation (substitution T to C)	Isoleucine→threonine at position 330
LD 054	Mutation (substitution C to T)	Alanine→threonine at position 80
LD 121	Mutation (substitution G to A)	Valine→isoleucine at position 36
LD 126	Mutation (substitution A to G)	No amino acid substitution
IR LD 130 to LD 131	Insertion (1 nt, A)	
IR LD 0135 to LD 136	Insertion (1 nt, A)	

a IR, intergenic region; LD, gene identifier.

### Data availability.

The genome sequence of the Neethling-RIBSP strain of the lumpy skin disease virus has been deposited in GenBank under accession number MT130502, and the raw data have been submitted to the SRA under BioProject number PRJNA615775.
